# Stability-Indicating RP-HPLC Method for Simultaneous Estimation of Enrofloxacin and Its Degradation Products in Tablet Dosage Forms

**DOI:** 10.1155/2015/735145

**Published:** 2015-01-29

**Authors:** V. Ashok Chakravarthy, B. B. V. Sailaja, Avvaru Praveen Kumar

**Affiliations:** ^1^Department of Inorganic and Analytical Chemistry, Andhra University, Visakhapatnam 530003, India; ^2^Department of Chemistry, Changwon National University, Changwon 641-773, Republic of Korea

## Abstract

The present work was the development of a simple, efficient, and reproducible stability-indicating reverse-phase high performance liquid chromatographic (RP-HPLC) method for simultaneous determination enrofloxacin (EFX) and its degradation products including ethylenediamine impurity, desfluoro impurity, ciprofloxacin impurity, chloro impurity, fluoroquinolonic acid impurity, and decarboxylated impurity in tablet dosage forms. The separation of EFX and its degradation products in tablets was carried out on Kromasil C-18 (250 × 4.6 mm, 5 *μ*m) column using 0.1% (v/v) TEA in 10 mM KH_2_PO_4_ (pH 2.5) buffer and methanol by linear gradient program. Flow rate was 1.0 mL min^−1^ with a column temperature of 35°C and detection wavelength was carried out at 278 nm and 254 nm. The forced degradation studies were performed on EFX tablets under acidic, basic, oxidation, thermal, humidity, and photolytic conditions. The degraded products were well resolved from the main active drug and also from known impurities within 65 minutes. The method was validated in terms of specificity, linearity, LOD, LOQ, accuracy, precision, and robustness as per ICH guidelines. The results obtained from the validation experiments prove that the developed method is a stability-indicating method and suitable for routine analysis.

## 1. Introduction

Enrofloxacin (EFX) (1-cyclopropyl-7-(4-ethylpiperazin-1-yl)-6-fluoro-4-oxo-1,4-dihydro quinoline-3-carboxylic acid) belongs to the group of synthetic 6-fluoroquinolones or 4-quinolones derived from the core structure of nalidixic acid. As a result of gradual changes to the basic molecule, antimicrobial properties were considerably increased and pharmacokinetics could be substantially improved, whereas the probability of adverse effects was reduced. Coplanar carbonyl groups (C=O) at positions 3 and 4 of the core structure are generally required for antimicrobial activity of the fluoroquinolones. They represent the binding site to the DNA gyrase complex. A fluorine atom, introduced at position 6, enhances the efficacy against Gram-negatives and broadens the spectrum against Gram-positive bacteria [1].

EFX is an amphoteric drug with pKa_1_ = 5.94 corresponding to carboxyl group and pKa_2_ = 8.70 corresponding to basic piperazinyl group and the isoelectric pH = 7.32 [2]. Due to the presence of carboxylic acid and amine functional groups (basic), the molecule has amphoteric and zwitter ionic properties which make EFX lipid soluble and enhance the ability to penetrate tissues, pus, and organic debris. The piperazine ring at position 7 further increases antimicrobial activity, especially against Pseudomonas organisms. The presence of a -C_2_H_5_ group which is attached to the piperazine ring enhances tissue penetration and decreases central nervous system toxicity by reducing drug binding to GABA receptors in the brain.

EFX is a pale or light yellow [3] colored crystalline substance with a high degree of purity. In water at pH 7, it is slightly soluble. However, as it contains acidic and basic groups (betaine structure), it can readily be brought into solution when the pH values are either alkaline or acidic. Liquid formulations of Baytril for parenteral administration contain freely soluble salts of EFX in an aqueous solution. Due to the high hydrolytic stability of the active ingredient, these solutions are very stable. The tablet formulations contain EFX in its original betaine form-1 [1, 4, 5]. In veterinary medicine it is administered by subcutaneous injection to cattle and intramuscular injection to pigs and orally to cattle, pigs, turkeys, and chickens, for the treatment of infections of the respiratory and alimentary tract [6].

From the literature survey it is evident that few methods were available for the determination of EFX and its impurities in tablet dosage forms by using high performance liquid chromatography (HPLC). Garcia et al. developed a method for the simultaneous determination of EFX and its primary metabolite ciprofloxacin in plasma by HPLC with fluorescence detection [7]. Souza et al. developed a HPLC method for determination of EFX [8]. Tyczkowska et al. developed high performance liquid chromatographic method for the simultaneous determination of EFX and its primary metabolite ciprofloxacin in canine serum and prostatic tissue [9]. Horie et al. developed simultaneous determination of benofloxacin, danofloxacin, enrofloxacin, and ofloxacin in chicken tissues by high performance liquid chromatography [10]. Idowu and Peggins developed simple, rapid determination of EFX and ciprofloxacin in bovine milk and plasma by HPLC with fluorescence detection [11]. The USP [12] and Eur. Ph. [3] have developed methods for the determination of EFX and its related impurities in drug substance by thin layer chromatography (TLC) and HPLC methods. TLC method was developed for the determination of fluoroquinolonic acid and HPLC method for the estimation of its two impurities, ciprofloxacin and desfluoro compound in both USP [12] and Ph. Eur. [3].

As per the literature review, no method was reported for the estimation of EFX and its degradation products in finished dosage forms by using HPLC. The present research work describes the simultaneous estimation of EFX and its degradation products in tablet dosage forms using HPLC. Methanol was used as solvent for the development and validation of this method as it is often less expensive and less toxic than acetonitrile. The work gives a sensitive, specific, and stability-indicating method for the determination of impurities of EFX in a single method by HPLC rather than performing two analytical techniques of HPLC and TLC. Time required for the TLC analysis, man power, and solvent consumption for performing TLC analysis can be saved and finally supporting towards green environment by following health safety and environment guidelines. Developed LC method was validated with respect to LOD, LOQ, linearity, precision, accuracy, and robustness. Forced degradation studies were carried out to verify the stability-indicating nature of the LC method.

## 2. Experimental

### 2.1. Chemicals and Reagents

Qualified standards (EFX purity ~99.5%, decarboxylated impurity ~99.6%, ethylenediamine impurity ~95.6%, desfluoro impurity ~99.3%, ciprofloxacin impurity ~99.2%, chloro impurity ~99.9%, and fluoroquinolonic acid impurity ~98.9%) and samples of EFX were obtained from local laboratories and were used without any further purification. HPLC grade methanol (MeOH purity ~99.7%) and acetonitrile (ACN purity ~99.8%) were obtained from Rankem (Mumbai, India). Orthophosphoric acid (purity ~85%) was received from Qualigens Fine Chemicals (Mumbai, India). Potassium dihydrogen orthophosphate (KH_2_PO_4_ purity ≥99.0%) was purchased from Merck specialties Pvt. Ltd. (Worli, Mumbai). Triethylamine (TEA purity ≥99.0%) was purchased from Spectrochem Pvt. Ltd. (Mumbai, India). Citric acid (purity ~99.5%) was obtained from Merck specialties Pvt. Ltd. (Worli, Mumbai).

### 2.2. Instrumentation

The Waters LC system (Milford, MA, USA) equipped with a diode array detector was used for method development and forced degradation studies. The output signal was monitored and processed using Empower software. Waters LC consists of 2695 separation modules and 2996 PDA detectors used for validation study. Intermediate precision was carried out using waters 2695 separation modules with 2487 dual wavelength detectors. Photolytic chamber was used for photolytic degradation and thermal degradation samples were kept at 80°C for 5 days in an oven.

### 2.3. Chromatographic Conditions

The chromatographic separation was achieved on a Kromasil C-18, 250 × 4.6 mm, 5 *μ*m column using mobile phase-A composed of 10 mM KH_2_PO_4_ containing 0.1% of TEA (v/v) (pH adjusted to 2.50 ± 0.05 with orthophosphoric acid) and mobile phase-B was MeOH. The mobile phase-A was filtered with 0.45 *μ*m nylon filter. Gradient program used for chromatographic separation was shown in Table 1. Flow rate was set to 1.0 mL min^−1^ with a column temperature of 35°C. Detection wavelength was carried out at 278 nm for ethylenediamine, desfluoro, ciprofloxacin, and chloro impurities and 254 nm for fluoroquinolonic acid and decarboxylated impurities. The injection volume was 10 *μ*L. Citrate buffer (pH 4.0) and MeOH in the ratio of 50 : 50 were used as diluent for the preparation of standards and samples. Citrate buffer was prepared by dissolving 2.0 g of citric acid and 0.5 g of potassium hydroxide in 1 liter of HPLC grade water and adjusted pH of the resultant solution to 4.00 ± 0.05 with dilute orthophosphoric acid.

### 2.4. Preparation of Standard and Sample Solutions

#### 2.4.1. Standard Stock Solution of EFX

We accurately weighed and transferred 50 mg of EFX working standard into a 100 mL volumetric flask. We added about 70 mL of diluent and sonicated it to dissolve with intermittent shaking. The resulting solution is diluted up to the mark with diluent and mixed well.

#### 2.4.2. Preparation of Standard Solution

We transferred 5 mL of EFX standard stock solution into a 50 mL volumetric flask and diluted it up to the mark with the diluent. We further diluted 5 mL of this solution into 50 mL with the diluent mixed well.

#### 2.4.3. Preparation of Sample Solution

We determined the average weight of 20 tablets and crushed to fine powder. We accurately weighed and transferred a sample powder equivalent to 50 mg of EFX into a 100 mL volumetric flask. We added about 70 mL of diluent and sonicated it for 60 min with intermittent shaking. We made up the volume of 100 mL volumetric flask with diluent and then filtered the solution through 0.45 *μ*m PVDF membrane filter.

### 2.5. Method Validation

#### 2.5.1. Specificity/Stress Studies

Specificity is the ability to assess unequivocally the analyte in the presence of components which may be expected to be present. Typically these might include impurities, degradants, matrix, and so forth. The specificity of the developed method was established to prove the absence of interference from placebo peaks (excipients) which is part of required pharmaceutical preparation. Degradation study was performed by subjecting the tablet powder to accelerated degradations such as acid, alkaline, oxidation, thermal, humidity, and photolytic conditions to evaluate the interference of degradation impurities. Thermal degradation was performed by keeping the placebo and tablets in different petri dishes and then placed them in an oven at 60°C for 3 days. Humidity degradation was performed by placing the tablet and placebo powders in two separate petri dishes and kept in a humidity chamber at 90% RH, 25°C for 7 days. Photolytic study was carried out by placing the placebo and tablets in separate petri dishes in a photolytic chamber at 1.2 million lux hour's illumination and 200-watt hours/square meter ultraviolet energy. Acid, base, and oxidation degradations were performed by adding 1 mL of 5 N HCl, 1 mL of 5 N NaOH, and 1 mL of 30% peroxide solution (H_2_O_2_), respectively, to the placebo and tablet powders at 70°C for 1 hour.

#### 2.5.2. LOD and LOQ

The detection limit of an individual analytical procedure is the lowest amount of analyte in a sample which can be detected but not necessarily quantitated as an exact value. The quantitation limit of an individual analytical procedure is the lowest amount of analyte in a sample which can be quantitatively determined with suitable precision and accuracy. The quantitation limit is a parameter of quantitative assays for low levels of compounds in sample matrices and is used particularly for the determination of impurities and/or degradation products. The limit of detection (LOD) and limit of quantitation (LOQ) were important for the impurity tests and the assays of dosages containing low drug levels. The LOD is generally quoted as the concentration yielding a signal-to-noise ratio of 2 : 1 or 3 : 1 and LOQ is quoted as the concentration yielding a signal-to-noise ratio of 10 : 1. The signal-to-noise ratio is determined by the following equation:

(1) $$\begin{array}{c} s=\frac{H}{h}, \end{array}$$

where *H* = height of the peak corresponding to the component. *h* = absolute value of the largest noise fluctuation from the baseline of the chromatogram of a blank solution.

LOD and LOQ are also determined based on the standard deviation of the response and the slope. The detection limit is expressed as “3.3*σ*/*S*” and quantification limit is expressed as “10*σ*/*S*” where *σ* is the standard deviation of the response and *S* is the slope of the calibration curve.

#### 2.5.3. Linearity

The linearity of an analytical procedure is its ability (within a given range) to obtain test results which are directly proportional to the concentration (amount) of analyte in the sample. Linearity is the ability of the method to obtain results which are either directly or after mathematical transformation proportional to the concentration of the analyte within a given range. The linearity of response for EFX and their related impurities were determined in the range from LOQ to 120%. The seven concentrations of each component were subjected to regression analysis by least-squares method to calculate correlation coefficient and calibration equation. The method of linear regression was used for the data evaluation.

#### 2.5.4. Precision

The precision of an analytical procedure expresses the closeness of agreement (degree of scatter) between a series of measurements obtained from multiple sampling of the same homogeneous sample under the prescribed conditions. Precision is considered at two levels: repeatability (method precision), intermediate precision. Precision should be investigated using homogeneous, authentic samples. Repeatability expresses the precision under the same operating conditions over a short interval of time. Repeatability is also termed intra-assay precision. Intermediate precision expresses within-laboratories variations: different days, different analysts, different equipment, and so forth. The precision was expressed as the relative standard deviation (RSD):

(2) $$\begin{array}{c} \%\text{RSD}=\left(\frac{\text{Standard}\;\;\text{deviation}}{\text{average}}\right)\times 100. \end{array}$$

Precision and intermediate precision of the developed method were carried out by 6 determinations (preparations) of the test solution by injecting the impurities spiked solution and calculated the %RSD for each impurity.

#### 2.5.5. Accuracy

The accuracy of an analytical procedure expresses the closeness of agreement between the value which is accepted as either a conventional true value or an accepted reference value and the value found. This is sometimes termed trueness. Accuracy or trueness was determined by applying the method to samples in which known amounts of analyte have been added. These should be analyzed against standard and blank solutions to ensure that no interference exists. The accuracy was calculated from the test results as a percentage of the analyte recovered by the assay.

Accuracy of the present method was carried out by injecting the impurities spiked solution at different concentration levels of LOQ, 100% and 120% to their specification limit, in triplicate determinations. The % recovery was calculated for each impurity. The mean percentage recovery was calculated.

#### 2.5.6. Robustness

The robustness of an analytical procedure is a measure of its capacity to remain unaffected by small but deliberate variations in method parameters and provides an indication of its reliability during normal usage. Robustness of the method indicates the reliability of an analysis to assess the system suitability parameters under the influence of small but deliberate variations in method parameters. It was performed by injecting the impurities spiked solution and the stressed degradation sample solutions by changing several parameters including different batch of the same column, flow rate, column temperature, and minor change in organic composition.

#### 2.5.7. Solution Stability

The control sample solution and the standard solution containing EFX were prepared as per the test procedure. All these solutions were stored at room temperature. The freshly prepared solutions and the solutions which were stored at room temperature up to 24 hours were injected at different time intervals. The % impurity obtained at initial was compared with the % impurity obtained at different time intervals.

## 3. Results and Discussion

### 3.1. Optimization of Chromatographic Conditions

**Figure 1:**
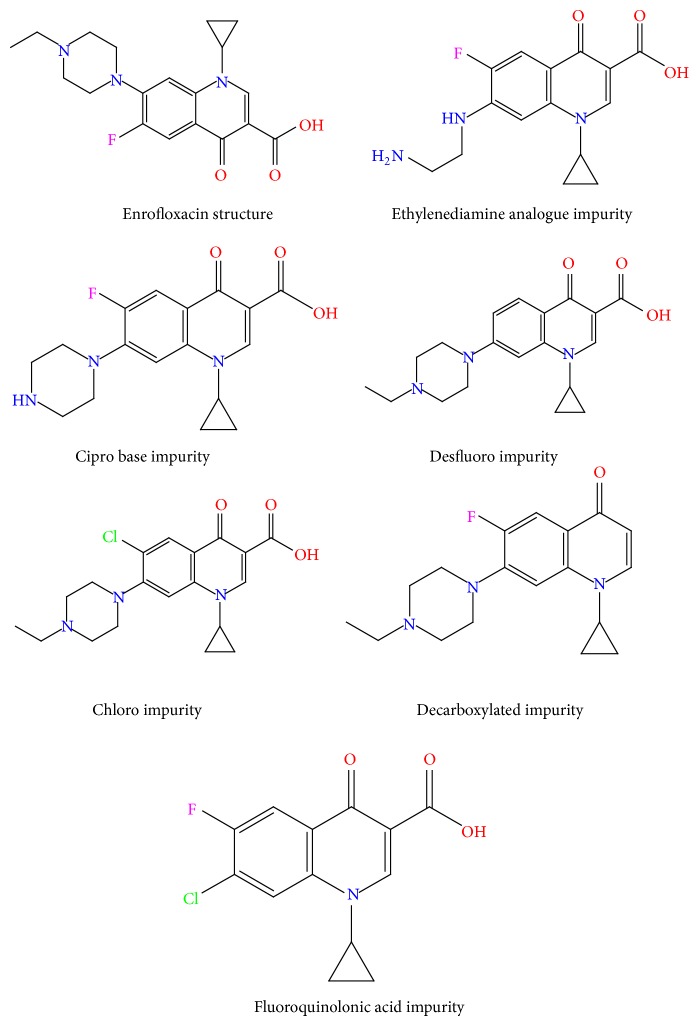
Structures of EFX and its related impurities.

The main purpose of the current chromatographic method was to develop a LC method for the separation and quantification of known and unknown degradation products of EFX in EFX tablets at trace level. EFX and its known impurities structures were shown in Figure 1. From the structure of EFX, it was observed that EFX has pKa_1_ = 5.94 corresponding to carboxyl group and pKa_2_ = 8.70 corresponding to basic piperazinyl group [2]. In spite of the fact that in reversed-phase separations, pH of selected buffer should have the pH ± 1.5 units from the pKa values of the analytes [13], the selection of buffer with proper pH leads to ionization of analytes which results in the sharp and symmetric peak shapes and reproducible retention times (RT). The pH of the mobile phase was selected at lower side as the pH increases silica dissolves slowly and results in inconsistent retention times and results. KH_2_PO_4_ has a wide range of pKa values; hence, initially we selected a buffer of 10 mM KH_2_PO_4_ composed of 0.5% TEA and set the pH of this solution to 3.00 ± 0.05 using orthophosphoric acid. ACN was used in the mobile phase along with Hypersil BDS C-18, 250 × 4.6 mm, 5 *μ*m column at a column temperature of 35°C. ACN was selected as solvent for initial method development trials as it produces sharp, symmetrical peaks with less column back pressure. TEA was used in the mobile phase to reduce tailing factor for EFX and its known impurities by reducing the silanol and sample interactions on the bonded surface of the HPLC column. Gradient program used for chromatographic separation was shown in Table 1. Placebo interference was observed at the retention time of decarboxylated impurity and broad peak shape observed for chloro impurity. Further trials were performed at a column temperature of 40°C and 50°C by using the same above chromatographic conditions; however placebo peak was not separated from the decarboxylated impurity.

Hence, mobile phase was changed to 10 mM KH_2_PO_4_ containing 0.1% of TEA (v/v) (pH adjusted to 2.50 ± 0.05 with orthophosphoric acid) and MeOH was used as solvent with Kromasil C-18, 250 × 4.6 mm, 5 *μ*m HPLC column at a column temperature of 40°C. MeOH was selected as solvent to separate decarboxylated impurity from placebo peak. Gradient program used for chromatographic separation was shown in Table 2. As a result, decarboxylated impurity was well separated from placebo peak and the resolution between the cipro base impurity and EFX peak is 1.6 only. Column temperature was changed to 45°C with slight change in gradient programme and remaining chromatographic conditions are unchanged. Gradient program used for chromatographic separation was shown in Table 3. Cipro base impurity was separated from the EFX peak but placebo interference was observed again at the retention time of decarboxylated impurity. Next trial run was carried out with slight change in linear gradient program at a column temperature of 40°C by keeping remaining chromatographic conditions the same as previous run. Gradient program used for chromatographic separation was shown in Table 4. Cipro base impurity was merged with EFX peak. Further trial was performed with the changes in gradient program with a column temperature of 35°C with all the chromatographic conditions the same as previous run. Gradient program used for chromatographic separation was shown in Table 5. All the impurities are well separated from each other and from EFX. Placebo peak was well separated from decarboxylated impurity with a resolution of 3.9. Hence, this method was finalized for separation of all the known impurities of EFX by using step gradient run.

The screening studies were performed on a variety of columns to cover a wide range of stationary phase properties including carbon chain length, carbon loading, and surface area. Each of the selected columns was screened with different mobile phase ratios, different column temperatures, and different type of organic solvents including MeOH and ACN. Kromasil C-18, 250 × 4.6 mm, 5 *μ*m column was selected for the final method due to reproducible results and better peak shapes. In most of the trials, major impurities of EFX are separated; however resolution between cipro base impurity and EFX is less and placebo peak interference with decarboxylated impurity is observed. The chromatograms of blank run are shown in Figures 3 and 4, chromatograms of placebo are shown in Figures 5 and 6, chromatograms of control sample (concentration ~0.5 mg mL^−1^) are shown in Figures 7 and 8, and chromatograms of 1% impurity spiked samples are shown in Figures 9 and 10.

The elution order of the impurities in different chromatographic conditions was Decarboxylated > ED analogue > Desfluoro > Cipro base > EFX > Chloro > FQ acid. In presence of ACN, ED analogue impurity was eluted first and desfluoro impurity was eluted next to the ED analogue impurity where as in presence of MeOH, desfluoro impurity was eluted first and ED analogue impurity was eluted next. Except for the above change, all the remaining impurities were eluted in the same order.

### 3.2. Selection of Wavelength for Impurities

**Figure 2:**
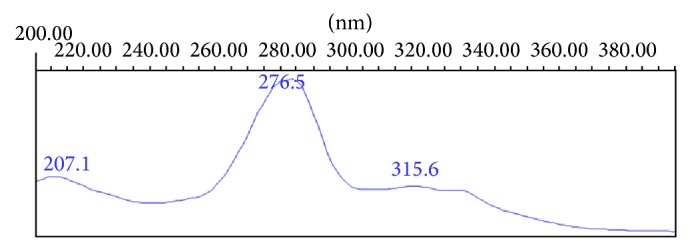
Spectra of EFX.

**Figure 3:**
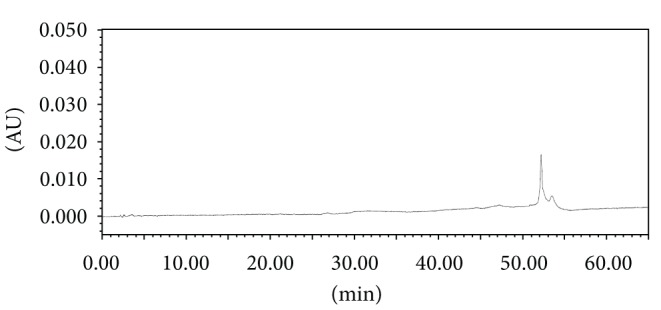
Blank run at 278 nm.

**Figure 4:**
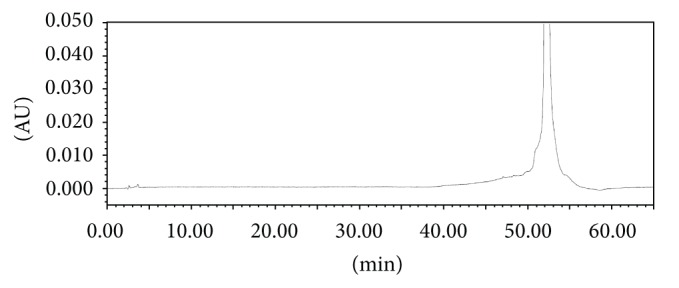
Blank run at 254 nm.

**Figure 5:**
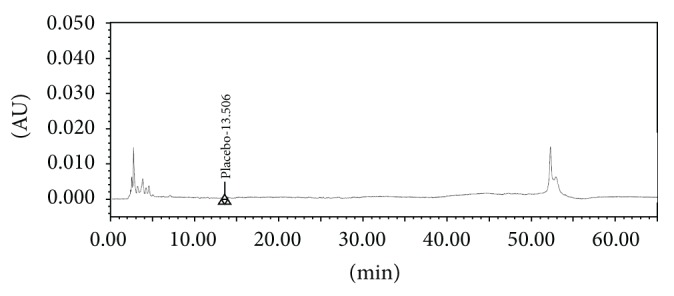
Placebo at 278 nm.

**Figure 6:**
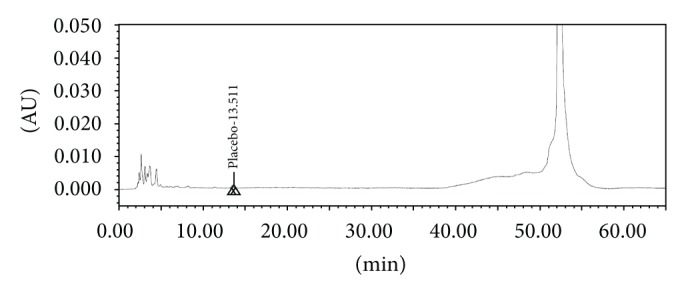
Placebo at 254 nm.

**Figure 7:**
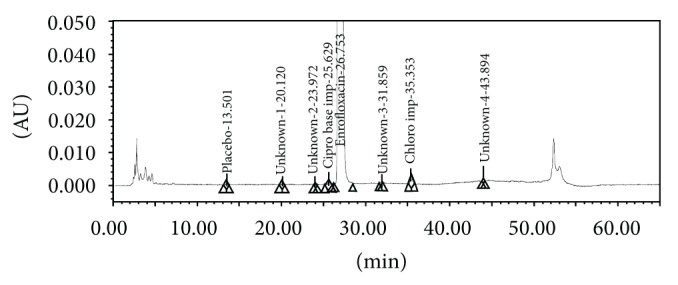
Control sample at 278 nm (concentration ~0.5 mg mL^−1^).

**Figure 8:**
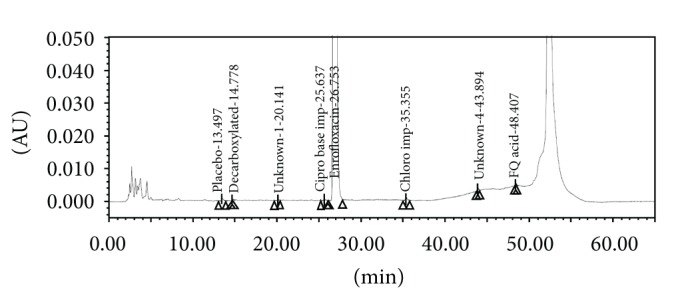
Control sample at 254 nm (concentration ~0.5 mg mL^−1^).

**Figure 9:**
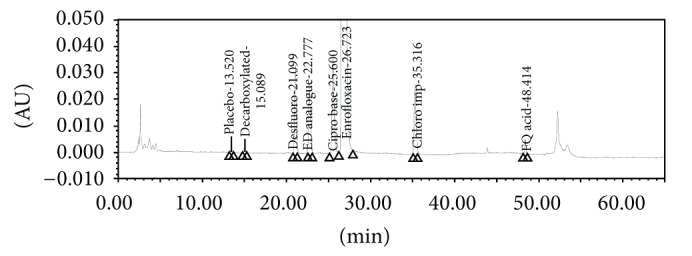
1% impurities spiked sample at 278 nm.

**Figure 10:**
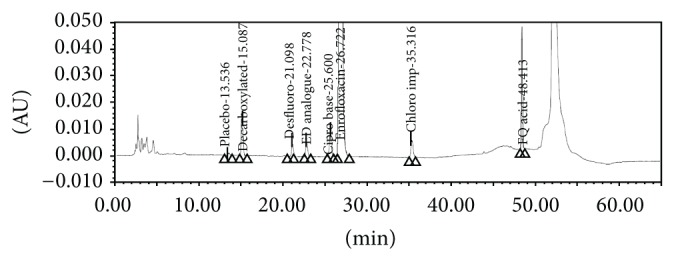
1% impurities spiked sample at 254 nm.

Spectra for all the known impurities and EFX were measured from 200 to 395 nm for wavelength maxima. The corresponding spectrum of EFX is shown in Figure 2. Based on the spectra maxima, 278 nm was selected for identification and quantification of ethylenediamine impurity, desfluoro impurity, ciprofloxacin impurity, and chloro impurity, and 254 nm was selected for identification and quantification of fluoroquinolonic acid impurity and decarboxylated impurities.

### 3.3. Optimization of Column Temperature

To study the temperature effect on resolution between the impurity peaks of EFX, we injected the impurities spiked solution at different column temperatures. It was observed that at a column temperature of 35°C, all the known degradation impurities were well separated when compared to the other column temperatures. The resolution between closely eluting cipro base impurity and EFX was found to be not less than 2.

### 3.4. Method Validation

The objective of validation of an analytical procedure is to demonstrate that it is suitable for its intended use. The described HPLC method has been extensively validated for its known degradation impurities and unknown impurities as per ICH guidelines [14].

After successful completion of method development [13, 15, 16], method validation [17–33] was performed to ensure that the developed method was capable of giving reproducible and reliable results when used by different operators employed on the same equipment of the same lab or of different laboratories. Stress testing needs to be performed to elucidate the inherent stability characteristics of the active drug substance and also to prove the stability-indicating capability of the method. The developed HPLC method was validated to quantify the degradation impurities of EFX in its tablet dosage form by determining the parameters including specificity, LOD, LOQ, linearity, accuracy, precision, and robustness according to the ICH guidelines.

#### 3.4.1. Specificity

Specificity of the developed method was performed by injecting the stressed degradation samples and the degradation impurities spiked solutions. The degradation study was carried out using the samples which include (i) tablet powder containing EFX and (ii) placebo powder without active drug EFX.

EFX was found to be stable in all the degradation conditions except in oxidation degradation where slight degradation was observed. Spectral homogeneity of EFX and their known and unknown impurities was checked. Peak purity passed for both the main active and all the known impurities. Purity angle value was less than the purity threshold for all peaks indicating all peaks are spectrally homogeneous. Also spectral homogeneity of known impurities in degradation samples, found to be similar with those obtained for the individual impurities, suggests that no peak was being coeluted at the retention time of respective known impurities. The degradation results of EFX in various stress conditions were shown in Table 6. The results indicate EFX undergoes degradation in presence of oxidation condition to form major unknown impurity.

#### 3.4.2. LOD and LOQ

The LOD and LOQ were determined for EFX and their impurities by injecting a series of solutions with known concentrations. We calculated the *S*/*N* ratio for these solutions and selected the concentration at which level *S*/*N* was about 3 for LOD and the *S*/*N* ratio was about 10 for LOQ. *S*/*N* values of LOD and LOQ for EFX and their impurities were shown in Table 7.

#### 3.4.3. Linearity

The linear graphs were plotted between the peak areas versus concentration to obtain the calibration curve. The response obtained for all compounds was found to be linear from LOQ to 120% of standard concentration. The correlation coefficient found for all compounds was not less than 0.99. The relative response factor for EFX and all the impurities was determined against their respective standard and presented in Table 7. Statistical values of all compounds were shown in Table 7. The results demonstrate an excellent correlation between the peak area and concentration of all impurities.

#### 3.4.4. Precision

Method precision was determined by injecting the impurities spiked solution of six determinations and the observed values of %RSD were shown in Table 7. The %RSD for all compounds in impurities spiked solution for six determinations was not more than 1.9%. The intermediate precision of the method was studied by injecting the impurities spiked solution of six determinations and the values were shown in Table 7. The %RSD difference between the two analysts is less than 0.6%. Less difference between the two analysts shows that the developed method is precise and has good intermediate precision.

#### 3.4.5. Accuracy

The percentage recovery results for impurities of EFX were varied from 91.2% to 106.7% at three different concentration levels and the results were shown in Table 8. Based on the % recovery data, we concluded that the developed method is capable of the estimation of its related substances and is adequate for routine analysis.

#### 3.4.6. Robustness

In all the robust conditions (flow rate, column temperature, and organic composition change in mobile phase and columns) the resolution between two critical pairs (resolution between cipro base impurity and EFX) was not less than 1.8. Relative retention times (RRT) and resolution values for different robustness parameters were shown in Tables 9 and 10. Also the resolution between the remaining impurities from analytes was not significantly affected and elution pattern of the impurities remained unchanged. The peak shape for all the impurities was found to be good. Peak purity for all impurities also tested to observe no placebo peaks interference in all the robust conditions.

#### 3.4.7. Solution Stability

The impurity percent difference was determined for control sample solutions and percent difference was determined for EFX standard solution stored at room temperature in different time intervals up to 24 hours. All the impurities and standard solution were found to be stable up to 24 hours at room temperature. Solution stability results of EFX standard solution and impurities in control sample at room temperature were shown in Table 11.

## 4. Conclusions

A novel RP-HPLC method was developed for the separation and quantification of EFX and its related degradation impurities in its pharmaceutical dosage forms. Degradation behavior of EFX was studied under various degradation conditions. Unknown degradation impurity of 0.5% was formed from EFX in oxidation degradation and no degradation peaks were observed in other stress conditions. All the known degradation impurities and the unknown degradation impurities were well separated from EFX revealing the stability-indicating capability of the method. The developed method can be used for the quantification of related substances of EFX in routine analysis.

## Figures and Tables

**Table 1:** Gradient program.

Time	Buffer	Acetonitrile
0	87	13
34	87	13
46	20	80
52	20	80
55	87	13
60	87	13

**Table 2:** Gradient program.

Time	Buffer	Methanol
0	80	20
20	70	30
40	20	80
50	20	80
55	80	20
60	80	20

**Table 3:** Gradient program.

Time	Buffer	Methanol
0	80	20
20	75	25
40	15	85
50	15	85
55	80	20
65	80	20

**Table 4:** Gradient program.

Time	Buffer	Methanol
0	80	20
45	30	70
55	20	80
60	80	20
65	80	20

**Table 5:** Gradient program for developed method.

Time	Buffer	Methanol
0	88	12
35	66	34
45	30	70
50	20	80
55	88	12
65	88	12

**Table 6:** Degradation data of EFX tablets.

Degradation conditions	%Decarboxylated impurity	%ED analogue	%Ciprobase impurity	%Chloro impurity	%FQ acid	%Total unknown impurities	%Total impurities
Acid treatment (5 N HCl, 70°C, 1 hr)	—	0.02	0.05	0.12	0.03	0.10	0.31
Base treatment (5 N NaOH, 70°C, 1 hr)	0.04	—	0.05	0.12	0.02	0.14	0.37
Peroxide treatment (30% H_2_O_2_, 70°C, 1 hr)	0.04	—	0.06	0.12	0.02	0.62	0.86
Thermal-80°C, 5 days	—	—	0.05	0.12	0.02	0.11	0.29
Humidity-90% RH, 25°C, 7 days	0.01	—	0.06	0.12	0.02	0.13	0.33
Photolytic-1.2 m lux hr, 200 Watt hr/m^2^	—	—	0.06	0.13	0.01	0.17	0.38

**Table 7:** LOD, LOQ, linearity, relative response factor, and precision data.

Parameter	EFX at 278 nm	EFX at 254 nm	%Decarboxylated impurity	%FQ Acid impurity	%ED impurity	%Desfluoro impurity	%Cipro base impurity	%Chloro impurity
LOD (*μ*g mL^−1^)	0.106	0.266	0.105	0.100	0.099	0.103	0.106	0.106
*S*/*N* ratio	4	6	5	5	6	6	4	5
LOQ (*μ*g mL^−1^)	0.266	0.532	0.261	0.249	0.248	0.258	0.266	0.265
*S*/*N* ratio	13	17	15	17	19	18	14	15
Relative response factor	—	—	0.46	0.32	0.87	0.83	0.92	1.09
Method precision (% RSD)^a^	—	—	1.5%	1.6%	0.9%	1.8%	1.7%	1.9%
Intermediate precision (% RSD)^b^	—	—	1.8%	1.7%	1.3%	1.2%	1.6%	1.5%
*Regression statistics *								
Slope	71160	17902	39100	56718	82107	85531	77739	65090
Intercept	−481.8	−216.8	−1336.5	−1150.4	−445.7	−2163.6	−1660.2	2529.3
Coefficient of determination (*R* ^2^)	0.9937	0.9920	0.9966	0.9986	0.9888	0.9930	0.9990	0.9905
Intercept at 95% confidence interval (lower value–upper value)	6255.01–5291.4	1849.7–1416.1	3577.4–904.5	3336.2–1035.5	8949.5–8058.1	9364.1–5036.9	3963.5–643.1	3828.3–8886.8
Slope at 95% confidence interval (lower value–upper value)	64629.7–77689.3	16054.9–19748.9	36511.0–41687.9	54278.1–59158.2	72068.0–92146.6	77286.7–93775.9	75035.1–80443.2	57779.0–72400.9

^a^Method precision calculated from six preparations of impurities spiked solutions.

^b^Intermediate precision calculated from six preparations of impurities spiked solutions.

**Table 8:** Accuracy results.

%Impurity level	%Recovery range for triplicate preparations					
	%Decarboxylated impurity	%FQ Acid impurity	%ED impurity	%Desfluoro impurity	%Cipro base impurity	%Chloro impurity
LOQ	93.5–106.0	91.7–96.6	95.8–101.8	91.2–97.3	95.6–99.2	92.6–96.9
100%	98.2–104.6	95.8–98.5	94.7–97.0	93.6–96.3	94.9–97.7	95.2–100.5
120%	94.4–96.7	93.4–96.3	96.0–100.4	95.6–101.7	101.1–106.7	98.8–102.9

**Table 9:** Robustness results (control sample and column temperature variations).

Name of the impurity	Control sample		Using other batch columns		Low column temperature 30°C		High column temperature 40°C	
	RRT	Resolution	RRT	Resolution	RRT	Resolution	RRT	Resolution
%Decarboxylated impurity	0.57	—	0.56	—	0.56	—	0.56	—
%Desfluoro impurity	0.79	15.25	0.79	14.31	0.79	15.83	0.77	13.82
%ED impurity	0.85	3.92	0.85	3.58	0.86	4.17	0.83	3.12
%Cipro base impurity	0.96	6.17	0.96	5.57	0.96	5.62	0.94	6.13
Enrofloxacin	1.00	2.16	1.00	1.85	1.00	1.81	1.00	2.48
%Chloro impurity	1.32	16.28	1.32	13.78	1.31	15.69	1.33	15.71
%FQ acid impurity	1.81	36.71	1.84	36.64	1.71	28.86	1.93	39.78

**Table 10:** Robustness results (flow rate and minor component change variations).

Name of the impurity	Flow rate 1.1 mL/min		Flow rate 0.9 mL/min		Higher methanol concentration at 45 min to 72%		Lower methanol concentration at 45 min to 68%	
	RRT	Resolution	RRT	Resolution	RRT	Resolution	RRT	Resolution
%Decarboxylated impurity	0.55	—	0.57	—	0.54	—	0.58	—
%Desfluoro impurity	0.78	15.56	0.79	15.13	0.77	15.26	0.80	15.35
%ED impurity	0.85	3.66	0.85	4.05	0.84	3.88	0.85	3.47
%Cipro base impurity	0.95	6.14	0.96	6.00	0.95	5.77	0.95	5.76
Enrofloxacin	1.00	2.30	1.00	1.99	1.00	2.00	1.00	2.16
%Chloro impurity	1.19	16.27	1.31	15.82	1.34	15.63	1.30	15.40
%FQ acid impurity	1.85	38.26	1.76	33.95	1.93	39.33	1.70	29.50

**Table 11:** Solution stability results of standard and control sample at room temperature.

Name of the impurity	Time interval		
	Initial	After 24 hours	%Difference
%Assay of standard solution	99.7	99.4	0.3
%Decarboxylated impurity	0.01	0.01	0.00
%FQ acid impurity	0.01	0.01	0.00
%ED impurity	Not applicable	Not applicable	Not applicable
%Desfluoro impurity	Not applicable	Not applicable	Not applicable
%Cipro base impurity	0.06	0.05	0.01
%Chloro impurity	0.12	0.11	0.01
%Unknown impurity	0.05	0.05	0.00
